# Validation of the Virtual Reality Neuroscience Questionnaire: Maximum Duration of Immersive Virtual Reality Sessions Without the Presence of Pertinent Adverse Symptomatology

**DOI:** 10.3389/fnhum.2019.00417

**Published:** 2019-11-26

**Authors:** Panagiotis Kourtesis, Simona Collina, Leonidas A. A. Doumas, Sarah E. MacPherson

**Affiliations:** ^1^Human Cognitive Neuroscience, Department of Psychology, University of Edinburgh, Edinburgh, United Kingdom; ^2^Department of Psychology, University of Edinburgh, Edinburgh, United Kingdom; ^3^Lab of Experimental Psychology, Suor Orsola Benincasa University of Naples, Naples, Italy; ^4^Interdepartmental Centre for Planning and Research “Scienza Nuova”, Suor Orsola Benincasa University of Naples, Naples, Italy

**Keywords:** virtual reality, VRISE, VR sickness, cybersickness, neuroscience, neuropsychology, psychology, motion sickness

## Abstract

There are major concerns about the suitability of immersive virtual reality (VR) systems (i.e., head-mounted display; HMD) to be implemented in research and clinical settings, because of the presence of nausea, dizziness, disorientation, fatigue, and instability (i.e., VR induced symptoms and effects; VRISE). Research suggests that the duration of a VR session modulates the presence and intensity of VRISE, but there are no suggestions regarding the appropriate maximum duration of VR sessions. The implementation of high-end VR HMDs in conjunction with ergonomic VR software seems to mitigate the presence of VRISE substantially. However, a brief tool does not currently exist to appraise and report both the quality of software features and VRISE intensity quantitatively. The Virtual Reality Neuroscience Questionnaire (VRNQ) was developed to assess the quality of VR software in terms of user experience, game mechanics, in-game assistance, and VRISE. Forty participants aged between 28 and 43 years were recruited (18 gamers and 22 non-gamers) for the study. They participated in 3 different VR sessions until they felt weary or discomfort and subsequently filled in the VRNQ. Our results demonstrated that VRNQ is a valid tool for assessing VR software as it has good convergent, discriminant, and construct validity. The maximum duration of VR sessions should be between 55 and 70 min when the VR software meets or exceeds the parsimonious cut-offs of the VRNQ and the users are familiarized with the VR system. Also, the gaming experience does not seem to affect how long VR sessions should last. Also, while the quality of VR software substantially modulates the maximum duration of VR sessions, age and education do not. Finally, deeper immersion, better quality of graphics and sound, and more helpful in-game instructions and prompts were found to reduce VRISE intensity. The VRNQ facilitates the brief assessment and reporting of the quality of VR software features and/or the intensity of VRISE, while its minimum and parsimonious cut-offs may appraise the suitability of VR software for implementation in research and clinical settings. The findings of this study contribute to the establishment of rigorous VR methods that are crucial for the viability of immersive VR as a research and clinical tool in cognitive neuroscience and neuropsychology.

## Introduction

Immersive virtual reality (VR) has emerged as a novel tool for neuroscientific and neuropsychological research (Bohil et al., 2011; Parsons, 2015; Parsons et al., 2018). Nevertheless, there are concerns pertinent to implementing VR in research and clinical settings, especially regarding the head-mounted display (HMD) systems (Sharples et al., 2008; Bohil et al., 2011; de França and Soares, 2017; Palmisano et al., 2017). A primary concern is the presence of adverse physiological symptoms (i.e., nausea, dizziness, disorientation, fatigue, and postural instability), which are referred to as motion-sickness, cybersickness, VR sickness or VR induced symptoms and effects (VRISE) (Sharples et al., 2008; Bohil et al., 2011; de França and Soares, 2017; Palmisano et al., 2017).

Longer durations in a virtual environment have been associated with a higher probability of experiencing VRISE, while the intensity of VRISE also appears to increase proportionally with the duration of the VR session (Sharples et al., 2008). However, extensive linear and angular accelerations provoke intense VRISE, even in a short period of time (McCauley and Sharkey, 1992; LaViola, 2000; Gavgani et al., 2018). VRISE may place the health and safety of the participants or patients at risk of experiencing adverse physiological symptoms (Parsons et al., 2018). Research has also shown that VRISE induce significant decreases in reaction times and overall cognitive performance (Nalivaiko et al., 2015; Nesbitt et al., 2017; Mittelstaedt et al., 2019), as well as substantially increasing body temperatures and heart rates (Nalivaiko et al., 2015), which may compromise physiological data acquisition. Furthermore, the presence of VRISE has been found to significantly augment cerebral blood flow and oxyhemoglobin concentration (Gavgani et al., 2018), electrical brain activity (Arafat et al., 2018), and the connectivity between stimulus-response regions and nausea-processing regions (Toschi et al., 2017). Thus, VRISE appear to confound the reliability of neuropsychological, physiological, and neuroimaging data (Kourtesis et al., 2019).

To our knowledge, there do not appear to be any guidelines as to the appropriate maximum duration of VR research and clinical sessions to evade or alleviate the presence of VRISE. Recently, our work has suggested that VRISE are substantially reduced or prevented by VR software that facilitates ergonomic navigation (e.g., physical movement) and interaction (e.g., direct-hand tracking) facilitated by the hardware capabilities (e.g., motion tracking) of commercial, contemporary VR HMDs comparable to or more advanced than the HTC Vive and/or Oculus Rift (Kourtesis et al., 2019). However, there are other factors such as the type of display and its features that may also induce or reduce VRISE (Mittelstaedt et al., 2018; Kourtesis et al., 2019). Nevertheless, we note that adequate technological competence is required to be able to implement appropriate VR hardware and/or software. In an attempt to reach a methodological consensus, we have proposed minimum hardware and software features, which appraise the suitability of VR hardware and software (see Table 1; Kourtesis et al., 2019).

**TABLE 1:** Domains and criteria for VR research/clinical software.

**Domains**	**User experience**	**Game mechanics**	**In-game assistance**	**VRISE**
CRITERIA	An adequate level of immersion	A suitable navigation system (e.g., teleportation)	Digestible tutorials	Absence or insignificant presence of nausea
	Pleasant VR experience	Availability of physical movement	Helpful tutorials	Absence or insignificant presence of disorientation
	High quality graphics	Naturalistic picking/placing of items	Adequate duration of tutorials	Absence or insignificant presence of dizziness
	High quality sounds	Naturalistic use of items	Helpful in-game instructions	Absence or insignificant presence of fatigue
	Suitable hardware (HMD and computer)	Naturalistic 2-handed interaction	Helpful in-game prompts	Absence or insignificant presence of instability

*Derived from Kourtesis et al. (2019).*

While VRISE may occur for various reasons, they are predominantly the undesirable outcomes of hardware and software insufficiencies (e.g., low resolution and refresh rates of the image, a narrow field of view, non-ergonomic interactions, and inappropriate navigation modes) (de França and Soares, 2017; Palmisano et al., 2017; Kourtesis et al., 2019). In terms of hardware, the technical specifications of the computer (e.g., processing power and graphics card), and VR HMD (e.g., the field of view, refresh rate, and resolution) suffice to appraise their suitability (Kourtesis et al., 2019). However, there is not a tool to quantify the software’s recommended features, as well as the intensity of VRISE (Kourtesis et al., 2019). Currently, the most frequently used measure of VRISE is the simulator sickness questionnaire (SSQ), which only considers the symptoms pertinent to simulator sickness (Kennedy et al., 1993). However, the SSQ does not assess software attributes (Kennedy et al., 1993), and there is an argument that simulator sickness symptomatology may not be identical to VRISE (Stanney et al., 1997). There is thus a need for a tool, which will enable researchers to assess both the suitability of VR software, as well as the intensity of VRISE.

Our recent technological literature review of VR hardware and software pinpointed four domains that should be considered in the development or selection of VR research/clinical software (Kourtesis et al., 2019). The domains are user experience, game mechanics, in-game assistance, and VRISE. Each domain has five criteria that should be met to ensure the appropriateness of the software (see Table 1). Also, in the same study, the meta-analysis of 44 VR neuroscientific studies revealed that most of the studies did not report quantitatively VR software’s quality and/or VRISE intensity (Kourtesis et al., 2019). In an attempt to provide a brief tool for the appraisal of VR research/clinical software features and VRISE intensity, we developed the virtual reality neuroscience questionnaire (VRNQ), which includes twenty questions that address five criteria under each domain. This study aimed to validate the VRNQ and provide suggestions for the duration of VR research/clinical sessions. We also considered the gaming experience of the participants to examine whether this may affect the duration of the VR sessions. Lastly, we investigated the software predictors of VRISE as measured by the VRNQ.

## Materials and Methods

### Participants

Forty participants (21 males) aged between 28 and 43 years (*M* = 32.08; *SD* = 3.54) and an educational level between 12 and 16 full-time years of education (*M* = 14.25; *SD* = 1.37) were recruited for the study. Eighteen participants (10 males) identified themselves as gamers through self-report and 22 as non-gamers (11 males). The gamer experience was a dichotomous variable (i.e., gamer or non-gamer) based on the participants’ response to a question asking whether they played games on a weekly basis. The participants responded to a call disseminated through mailing lists at the University of Edinburgh and social media. The study was approved by the Philosophy, Psychology and Language Sciences Research Ethics Committee of the University of Edinburgh. All participants provided written informed consent prior to taking part.

### Material

#### Hardware

An HTC Vive HMD with two lighthouse-stations for motion tracking was used with two HTC Vive’s wands with 6 degrees of freedom (DoF) to facilitate navigation and interactions within the environment (Kourtesis et al., 2019). The VR area where the participants were immersed and interacted with the virtual environments was 4.4 m^2^. Additionally, the HMD was connected to a laptop with an Intel Core i7 7700HQ processor at 2.80 GHz, 16 GB RAM, a 4095 MB NVIDIA GeForce GTX 1070 graphics card, a 931 GB TOSHIBA MQ01ABD100 (SATA) hard disk, and Realtek High Definition Audio.

#### Software

Three VR games were selected, which included ergonomic navigation (i.e., teleportation and physical mobility) and interactions (i.e., 6 DoF wands simulating hand movements) with the virtual environment. In line with Kourtesis et al. (2019), the VR software inclusion criteria (see Table 1) were: (1) ergonomic interactions which simulate real-life hand movements; (2) a navigation system which uses teleportation and physical mobility; (3) comprehensible tutorials pertinent to the controls; and (4) in-game instructions and prompts which assist the user in orientating and interacting with the virtual environment. The suitability of the VR software for both gamers and non-gamers was also considered. The selected VR games which met the above software criteria were: (1) “Job Simulator” (Session 1)^1^; (2) “The Lab” (Session 2)^2^; and (3) “Rick and Morty: Virtual Rick-ality” (Session 3)^3^. In “Job Simulator,” the participant becomes an employee who has several occupations, such as a cook (preparing simply recipes), car mechanic (doing rudimentary tasks e.g., replacing faulty parts), and an office worker (making calls and sending emails). In “The Lab,” the participant needs to complete several mini-games like slingshot (shooting down piles of boxes), longbow (shooting down invaders), xortex (spaceship-battles), postcards (visiting exotic places), human medical scan (exploring the human body), solar system (exploring the solar system), robot repair (repairing a robot), and secret shop (exploring a magical shop). In “Rick and Morty: Virtual Rick-ality,” the participant needs to complete several imaginary home-chores as in “Job Simulator,” though, in this case, the participant is required to follow a sequence of tasks according to a fictional storyline.

#### Virtual Reality Neuroscience Questionnaire (VRNQ)

The VRNQ measures the quality of user experience, game mechanics, and in-game assistance, as well as the intensity of VRISE. The VRNQ involves 20 questions where each question corresponds to one of the criteria for appropriate VR research/clinical software (e.g., the level of immersion; see Table 1). The 20 questions are grouped under four domains, where each domain encompasses five questions. Hence, VNRQ produces a total score corresponding to the overall quality of VR software, as well as four sub-scores (i.e., user experience, game mechanics, in-game assistance, VRISE). The user experience score is based on the intensity of the immersion, the level of enjoyment, as well as the quality of the graphics, sound, and VR technology (i.e., internal and external hardware). The game mechanics’ score depends on the ease to navigate, physically move, and interact with the virtual environment (i.e., use, pick and place, and hold items; two-handed interactions). The in-game assistance score appraises the quality of the tutorial(s), in-game instructions (e.g., description of the aim of the task), and prompts (e.g., arrows showing the direction). The VRISE are evaluated by the intensity of primary adverse symptoms and effects pertinent to VR (i.e., nausea, disorientation, dizziness, fatigue, and instability). VRNQ responses are indicated on a 7-point Likert style scale, ranging from 1 = extremely low to 7 = extremely high. The higher scores indicate a more positive outcome; this also applies to the evaluation of VRISE intensity. Hence, the higher VRISE score indicates a lower intensity of VRISE (i.e., 1 = extremely intense feeling, 2 = very intense feeling, 3 = intense feeling, 4 = moderate feeling, 5 = mild feeling, 6 = very mild feeling, 7 = absent). The VRNQ also includes space under each question, where the participant may provide optional qualitative feedback. For further details, please see the VRNQ in Supplementary Material.

### Procedure

The participants individually attended three separate VR sessions; in each session, they were immersed in different VR software. The period between each session was 1 week for each participant (i.e., 3 weeks in total). The participants went through an induction pertinent to the VR software for that session and the specific HMD and controllers used (i.e., HTC Vive and its 6DoF wands-controllers) before being immersed. Subsequently, the participants were asked to play the respective VR game until they completed it, or they felt any discomfort or fatigue. The duration of each VR session was recorded from the time the software was started until the participant expressed that they wanted to discontinue. At the end of each session, participants were asked to complete the VRNQ. The “Job Simulator” was always used in the 1st session, “The Lab” was always used in the 2nd session, and “Rick and Morty: Virtual Rick-ality” was always used in the 3rd session.

### Statistical Analyses

A reliability analysis of the VRNQ was conducted to calculate Cronbach’s alpha and inspect whether the items have adequate internal consistency for research and clinical purposes. A Cronbach’s alpha of 0.70–1.00 indicates good to excellent internal consistency (Nunally and Bernstein, 1994). A confirmatory factor analysis (CFA) was performed to examine the construct validity of the VRNQ in terms of convergent and discriminant validity (Cole, 1987). The reliability analysis and CFA were conducted using AMOS (version 24) (Arbuckle, 2014), and IBM Statistical Package for the Social Sciences (SPSS) 24.0 (Ibm Corp, 2016). Several tests for goodness of fit were implemented to allow the evaluation of VRNQ’s structure. The (CFI), Tuckere Lewis index (TLI), standardized root mean square residual (SRMR), and the root mean squared error of approximation (RMSEA) were used to assess model fit. A CFI and TLI equal to or greater than 0.90 indicate good structural model fit to the data (Hu and Bentler, 1999; Jackson et al., 2009; Hopwood and Donnellan, 2010). An SRMR and RMSEA less than 0.08 postulate a good fit to the data (Hu and Bentler, 1999; Hopwood and Donnellan, 2010). Lastly, the variance of the results was assessed by dividing the χ^2^ by the degrees of freedom (df), which is an indicator of the sample distribution (Hu and Bentler, 1999; Jackson et al., 2009; Hopwood and Donnellan, 2010).

The reliability and confirmatory factor analyses were conducted based on 120 observations (40 participants ^∗^ 3 sessions with different software). The *a priori* sample size calculator for structural equation models was used to calculate the minimum sample size for model structure. This calculator uses the error function formula, the lower bound sample size formula for a structural equation model, and the normal distribution cumulative distribution function (Soper, 2019a), which are in perfect agreement with the recommendations for statistical power analysis for the behavioral sciences (Cohen, 2013). A sample size of 100 observations was suggested as the minimum for conducting CFA to examine the model structure with statistical power equal to or greater than 0.80. Hence, the 120 observations in our sample appear adequate to conduct a CFA with statistical power equal to or greater than 0.80.

Bayesian Pearson correlation analyses were conducted to examine whether any of the demographic variables were significantly associated with the VRNQ total score and sub-scores, or the length of the VR sessions. Bayesian paired samples *t*-tests were performed to investigate possible differences between each session’s duration, as well as the VRNQ results for each VR game. Also, a Bayesian independent samples *t*-test examined whether there were any differences between gamers and non-gamers in the duration of the session. Lastly, a Bayesian linear regression was performed to examine the predictors of VRISE, where the Jeffreys–Zellner–Siow (JZS) mixed g-prior was used for the selection of the best model. JZS has the computational advantages of a g-prior in conjunction with the theoretical advantages of a Cauchy prior, which are valuable in variable selection for the best model (Liang et al., 2008; Rouder and Morey, 2012). For all the analyses, a Bayes Factor (BF_10_) ≥ 10 was set for statistical inference, which indicates strong evidence in favor of the alternative hypothesis (Rouder and Morey, 2012; Wetzels and Wagenmakers, 2012; Marsman and Wagenmakers, 2017). All the Bayesian analyses were performed using JASP (Version 0.8.1.2) (Jasp Team, 2017). The Bayesian Pearson correlation analyses and Bayesian linear regression analysis were conducted based on 120 observations (40 participants ^∗^ 3 different software sessions). The *post hoc* statistical power calculator was used to calculate the observed power of the best model using Bayesian linear regression analysis (Soper, 2019b).

## Results

### Reliability Analysis and CFA

**FIGURE 1:**
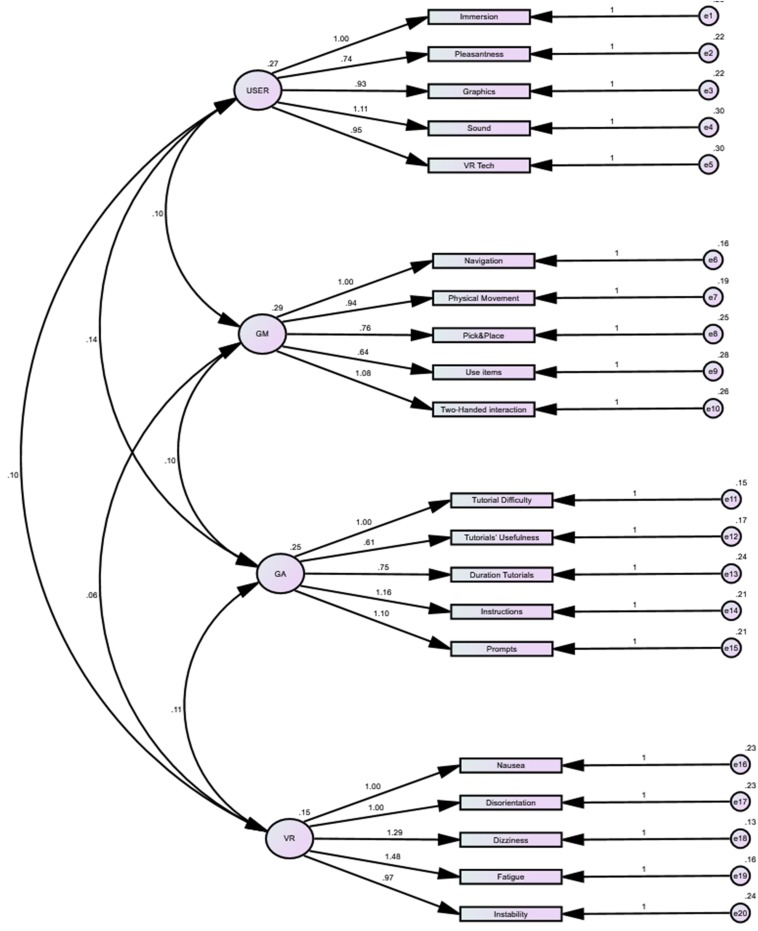
CFA: model’s path diagram. From left to right: the structural model illustrates the associations between VRNQ domains (paths with double headed arrow) and between each VRNQ domain and its items. At the right there are the error items (e) for each item; USER, user experience; GM, game mechanics; GA, in-game assistance; VR, VRISE.

The reliability analysis demonstrated good to excellent Cronbach’s α for each domain of the VRNQ (i.e., user experience – α = 0.89, game mechanics – α = 0.89, in-game assistance – α = 0.90, VRISE – α = 0.89; see Table 2), which indicate very good internal reliability (Nunally and Bernstein, 1994). VRNQ’s fit indices are displayed in Table 2 with their respective thresholds. The χ^2^/df was 1.61, which indicates good variance in the sample (Hu and Bentler, 1999; Jackson et al., 2009; Hopwood and Donnellan, 2010). Both CFI and TLI were close to 0.95, which suggest a good fit for the VRNQ model (Hu and Bentler, 1999; Jackson et al., 2009; Hopwood and Donnellan, 2010). Comparably, SPMR and RMSEA values were between 0.06 and 0.08, which also support a good fit (Hu and Bentler, 1999; Jackson et al., 2009; Hopwood and Donnellan, 2010). The VRNQ’s path diagram is displayed in Figure 1, where from left to right are depicted the correlations among the factors/domains of the VRNQ, the correlations between each factor/domain and its items, and the error terms for each item. The VRNQ items/questions are efficiently associated with their respective factor/domain, which shows good convergent validity (Cole, 1987). Furthermore, there was not any significant correlation amongst the factors/domains, which indicates good discriminant validity (Cole, 1987).

**TABLE 2:** Internal reliability and goodness of fit for the VRNQ.

**Statistics**	**Thresholds**	**Results**
Cronbach’s α	≥0.70	USER – 0.886 GM – 0.888 GA – 0.895 VR – 0.891
χ ^2^/df	≤2.00	1.610
Comparative fit index (CFI)	≥0.90	0.954
Tuckere Lewis index (TLI)	≥0.90	0.938
Standardized root mean square residual (SRMR)	<0.08	0.076
Root mean square error of approximation (RMSEA)	≤0.08	0.071

*VRNQ Domains: USER, user experience; GM, game mechanics; GA, in-game assistance; VR VRISE.*

### Descriptive Statistics of Sessions’ Duration and VRNQ Scores

**FIGURE 2:**
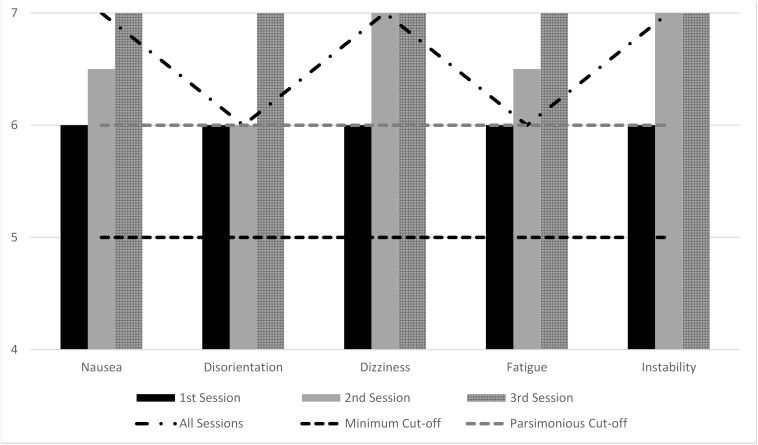
VRISE intensity in VR sessions as measured by VRNQ. Median scores of VRISE items of VRNQ; VRNQ Minimum Cut-off (≥); VRNQ Parsimonious Cut-off (≥); 1, Extreme intense feeling; 2, Very intense feeling; 3, Intense feeling; 4, Moderate feeling; 5, Mild feeling; 6, Very mild feeling; 7, Absent feeling.

The descriptive statistics for the sessions’ durations and the VRNQ scores are displayed in Table 3. In session 1, the participants were immersed for 59.65 (8.42) minutes. In session 1, the average time of gamers seems more than the average time of non-gamers (Table 3). In session 2, the participants spent 64.72 (6.24) minutes (Table 3). In session 3, gamers spent 70.44 (7.78) minutes, while non-gamers spent 65.73 (6.75) minutes (Table 3). The average total score of the VRNQ for all software was 126.30 (7.55) (maximum score is 140), where gamers and non-gamers scores did not appear to differ. Similarly, the median scores for each domain were 30–32 out of 35, where again gamers and non-gamers scores did not appear to differ. Importantly, all the VRISE scores (per item) for both gamers and non-gamers were equal to 5 (i.e., mild feeling), or 6 (i.e., very mild feeling), or 7 (absent feeling). The vast majority of scores were equal to 6 (i.e., very mild feeling) or 7 (absent feeling) (see Figure 2).

**TABLE 3:** Descriptive statistics: duration of VR sessions and VRNQ scores.

	**Group**	**N**	**Mean (SD)**	**SE**
Total duration	Gamers	18	199.39 (13.63)	3.21
	Non-Gamers	22	186.36 (11.76)	2.51
	Total	40	192.2 (14.09)	2.23
Duration of session 1	Gamers	18	65.61 (7.14)	1.68
	Non-Gamers	22	54.77 (5.91)	1.26
	Total	40	59.65 (8.42)	1.33
Duration of session 2	Gamers	18	63.33 (6.16)	1.45
	Non-Gamers	22	65.86 (6.21)	1.32
	Total	40	64.72 (6.24)	0.99
Duration of session 3	Gamers	18	70.44 (7.78)	1.83
	Non-Gamers	22	65.73 (6.75)	1.44
	Total	40	67.85 (7.52)	0.69
VRNQ total score out of 140 (across 3 sessions)	Gamers	18	127.2 (7.32)	0.99
	Non-Gamers	22	125.6 (7.71)	0.95
	Total	40	126.3 (7.55)	0.69
User’s experience (across 3 sessions) out of 35	Gamers	18	31.37 (2.73)	0.34
	Non-Gamers	22	30.91 (2.73)	0.37
	Total	40	31.12 (2.73)	0.25
Game mechanics (across 3 sessions) out of 35	Gamers	18	31.50 (2.68)	0.37
	Non-Gamers	22	31.32 (2.61)	0.32
	Total	40	31.40 (2.63)	0.24
In-game assistance (across 3 sessions) out of 35	Gamers	18	31.70 (2.59)	0.35
	Non-Gamers	22	31.65 (2.52)	0.31
	Total	40	31.68 (2.54)	0.23
VRISE (across 3 sessions) out of 35	Gamers	18	32.67 (2.17)	0.30
	Non-Gamers	22	31.71 (2.56)	0.32
	Total	40	32.14 (2.43)	0.22

### Minimum and Parsimonious Cut-Off Scores of VRNQ

Cut-off scores were calculated for the VRNQ total score and sub-scores to inspect the suitability of the assessed VR software (see Table 4). In the VRNQ, the ordinal 1–3 responses are paired with negative qualities, response 4 is paired with neutral/moderate qualities, and 5–7 responses are paired with positive qualities (see Supplementary Material). The minimum cut-offs suggest that if the median of the responses is 25 for every sub-score, and 100 in the total score (i.e., at least a median of 5 for every item), then the VRNQ outcomes indicate that the evaluated VR software is of an adequate quality not to cause any significant VRISE. Furthermore, the parsimonious cut-offs suggest that, if the median of the responses is 30 for every sub-score, and 120 for the total score (i.e., at least a median of 6 for every item) then the utilization of the parsimonious cut-offs more robustly supports the suitability of the VR software. The minimum and parsimonious cut-offs hence appear adequate to guarantee the safety, pleasantness, and appropriateness of the VR software for research and/or clinical purposes.

**TABLE 4:** VRNQ cut-offs.

**Score**	**Minimum cut-offs**	**Parsimonious cut-offs**
User experience	≥25/35	≥30/35
Game mechanics	≥25/35	≥30/35
In-game assistance	≥25/35	≥30/35
VRISE	≥25/35	≥30/35
VRNQ total score	≥100/140	≥120/140

*The median of each sub-score and totals scores should meet the suggested cut-offs to support that the evaluated VR software has an adequate quality without any significant VRISE. The utilization of the parsimonious cut-offs more robustly supports the suitability of the VR software.*

### Bayesian *T*-Tests

The Bayesian independent samples *t*-test between gamers and non-gamers indicated that the former spent significantly more time in VR across the total duration for the 3 sessions (BF_10_ = 14.99), as well as the duration of the 1st session (BF_10_ = 2,532; see Table 4) (Wetzels and Wagenmakers, 2012; Marsman and Wagenmakers, 2017). The difference is much smaller in the total duration than the difference in the 1st session. Thus, the difference between the gamers and non-gamers in the total duration appears to be driven by the substantial difference in the1st session’s duration (see Table 5). Conversely, the Bayesian paired samples *t*-test (i.e., differences between the VR games) indicated significant differences in the total score and every sub-score of VRNQ (see Table 6) between the VR software. The VR software in the 3rd session was evaluated higher than the VR software in the 1st and 2nd sessions, while the VR software in the 2nd session was rated better than the VR software in the 1st session. There was also an important difference between the duration of the 3rd session (longer) and the duration of the 1st session (shorter; BF_10_ = 103,568), while there was not a substantial difference between the duration of the 2nd and 3rd sessions (BF_10_ = 2.78), as well as between the duration of 1st and 2nd sessions (BF_10_ = 7.05; see Table 6) (Wetzels and Wagenmakers, 2012; Marsman and Wagenmakers, 2017).

**TABLE 5:** Bayesian independent samples *t*-test: gamers against non-gamers.

**Variables**	**Significance**	**BF*10***	**Error %**
Age		0.323	0.006
Education		0.325	0.006
Total duration	**^∗^**	14.987	7.044e-6
Session 1 duration	**^∗∗∗^**	2531.886	7.491e-8
Session 2 duration		0.595	0.006
Session 3 duration		1.580	0.003
VRNQ total		0.425	0.007
User’s experience		0.359	0.006
Game mechanics		0.315	0.006
In-game assistance		0.315	0.006
VRISE		0.745	0.003

*BF_10_ = Bayes Factor; ^∗^ BF_10_ > 10, ^∗∗∗^ BF_10_ > 100.*

**TABLE 6:** Bayesian paired samples *t*-tests: differences between the VR software.

**Pairs**		**Significance**	**BF*10***	**Error %**
Session 2 duration	Session 1 duration		7.049	∼0.001
Session 3 duration	Session 2 duration		2.783	∼3.276e-4
Session 3 duration	Session 1 duration	**^∗∗∗^**	103568.858	NaN
S3 VRNQ total	S2 VRNQ total	**^∗∗∗^**	6.942e + 12	NaN
S3 VRNQ total	S1 VRNQ total	**^∗∗∗^**	3.520e + 20	NaN
S2 VRNQ total	S1 VRNQ total	**^∗∗∗^**	8.500e + 17	NaN
S3 VRISE	S2 VRISE	**^∗∗∗^**	22075.036	NaN
S3 VRISE	S1 VRISE	**^∗∗∗^**	1.322e + 10	NaN
S2 VRISE	S1 VRISE	**^∗∗∗^**	1.160e + 7	NaN
S3 in-game assistance	S2 in-game assistance	**^∗∗∗^**	207216.904	NaN
S2 in-game assistance	S1 in-game assistance	**^∗∗∗^**	1.197e + 7	NaN
S3 in-game assistance	S1 in-game assistance	**^∗∗∗^**	8.028e + 10	NaN
S3 game mechanics	S2 game mechanics	**^∗∗∗^**	274310.417	NaN
S2 game mechanics	S1 game mechanics	**^∗∗∗^**	4.883e + 14	NaN
S3 game mechanics	S1 game mechanics	**^∗∗∗^**	2.876e + 14	NaN
S3 user’s experience	S2 user’s experience	**^∗∗∗^**	2.873e + 7	NaN
S3 in-game assistance	S1 user’s experience	**^∗∗∗^**	2.597e + 7	NaN
S2 user’s experience	S1 user’s experience	**^∗∗∗^**	1.708e + 6	NaN

*BF_10_ = Bayes Factor; ^∗^ BF_10_ > 10, ^∗∗^ BF_10_ > 30, ^∗∗∗^ BF_10_ > 100; S1, Session 1; S2, Session 2; S3, Session 3.*

### Bayesian Pearson Correlation Analyses and Regression Analysis

**FIGURE 3:**
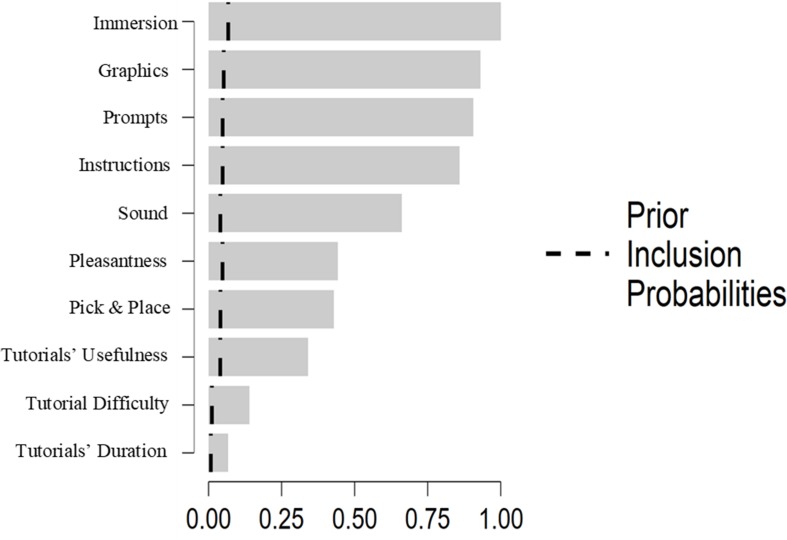
Variables’ prior inclusion Probabilities.

The Bayesian Pearson correlation analyses did not show any significant correlation between age and any of the VRNQ scores, between age and duration of the sessions, between education and any of the VRNQ scores, or between education and duration of the sessions. However, the duration of the session was positively correlated with the total VRNQ score [BF_10_ = 81.54; *r*(120) = 0.310, *p* < 0.001]. Furthermore, the VRISE score substantially correlated with the following VRNQ items: immersion, pleasantness, graphics, sound, pick and place, tutorial’s difficulty, tutorial’s usefulness, tutorial’s duration, instructions, and prompts (see Table 7). In contrast, VRISE did not significantly correlate with the following VRNQ items: VR tech, navigation, physical movement, use items, or two-handed interactions (see Table 7). Moreover, the Bayesian regression analysis indicated the five best models that predicted the VRNQ’s VRISE score (see Table 8). The best model includes the following items from the VRNQ: immersion, graphics, sound, instructions, and prompts. All the predictors exceeded the prior inclusion probabilities (see Figure 3). The best model showed a BF_M_ = 117.42, whereas the second-best model displayed a BF_M_ = 56.40 (see Table 8); hence, the difference between the best model compared to the second-best model was robust (Rouder and Morey, 2012; Wetzels and Wagenmakers, 2012; Marsman and Wagenmakers, 2017). Also, the best model has an *R*^2^ = 0.324 (see Table 8), which postulates that the model explains the 32.4% of the variance of VRISE score (Rouder and Morey, 2012; Wetzels and Wagenmakers, 2012). Lastly, the *post hoc* statistical power analysis for the best model indicated an observed statistical power of 0.998, *p* < 0.001, which postulates a high efficiency, precision, reproducibility, and reliability of the regression analysis and results (Button et al., 2013; Cohen, 2013).

**TABLE 7:** Bayesian Pearson correlations analyses: VRISE score with VRNQ items.

**Pairs**		**Significance**	**BF*10***	**r**
VRISE	Immersion	^∗∗∗^	1226.538	0.371
VRISE	Pleasantness	^∗^	20.504	0.273
VRISE	Graphics	^∗∗∗^	1629.195	0.377
VRISE	Sound	^∗∗∗^	18586.578	0.421
VRISE	VR Tech		5.094	0.228
VRISE	Navigation		4.808	0.226
VRISE	Physical movement		2.229	0.197
VRISE	Pick and place	^∗∗∗^	175.087	0.329
VRISE	Use items		0.405	0.109
VRISE	Two-handed interaction		0.506	0.123
VRISE	Tutorial difficulty	^∗∗∗^	28252.587	0.428
VRISE	Tutorials usefulness	^∗∗∗^	161.949	0.327
VRISE	Tutorials’ duration	^∗∗∗^	128.539	0.322
VRISE	Instructions	^∗∗∗^	952.871	0.366
VRISE	Prompts	^∗∗∗^	706510.726	0.476

*BF_10_ = Bayes Factor; ^∗^ BF_10_ > 10, ^∗∗^ BF_10_ > 30, ^∗∗∗^ BF_10_ > 100;*

**TABLE 8:** Models’ comparison: predictors of VRISE score.

**Models**	**P(M)**	**P(M| data)**	**BF_M_**	**BF_10_**	***R*^2^**
Prompts + Sound + Graphics + Immersion + Instructions	0.004	0.304	117.42^∗∗∗^	1.000	0.324
Prompts + Graphics + Immersion + Instructions + Pleasantness	0.004	0.173	56.47^∗∗^	0.571	0.317
Prompts + Sound + Graphics + Immersion + Instructions + Pick and Place	0.004	0.161	43.15^∗^	0.443	0.330
Prompts + Sound + Graphics + Immersion + Instructions + Pick and Place + Tutorials Usefulness + Pleasantness	0.021	0.123	6.62	0.072	0.337
Prompts + Graphics + Immersion + Instructions + Pick and Place + Tutorials Usefulness + Pleasantness	0.008	0.077	10.72^∗^	0.121	0.329

*P, Probability; M, Model; BF_*M*,_ Model’s Bayesian Factor; ^∗^ BF_*M*_ > 10, ^∗∗^ BF_*M*_ > 30, ^∗∗∗^ BF_*M*_ > 100; BF_10_ = BF against null model.*

## Discussion

### The VRNQ as a Research and Clinical Tool

The VRNQ is a short questionnaire (5–10 min administration time) which assesses the quality of VR software in terms of user experience, game mechanics, in-game assistance, and VRISE. The values of the fit indices of CFA (i.e., CFI, TLI, SPMR, and RMSEA) indicated that the VRNQ’s structure was a good fit to the data, which postulates good construct validity for the VRNQ (Hu and Bentler, 1999; Jackson et al., 2009; Hopwood and Donnellan, 2010). In addition, the construct validity of the VRNQ was supported by its convergent and discriminant validity (Cole, 1987). VRNQ items were strongly correlated with their grouping factor, which indicates robust convergent validity, while there were substantially poor correlations between the factors, which postulates very good discriminant validity (Cole, 1987). Furthermore, the Cronbach’s α for each VRNQ domain (i.e., user experience – α = 0.89, game mechanics – α = 0.89, in-game assistance – α = 0.90, VRISE – α = 0.89; see Table 2) suggest very good construct validity (Nunally and Bernstein, 1994). Henceforth, the VRNQ emerges as a valid and suitable tool to evaluate the quality of the VR research/clinical software as well as the intensity of the adverse VRISE.

Furthermore, minimum and parsimonious cut-off scores were calculated for the VRNQ total score and sub-scores to inspect the suitability of the assessed VR software. The minimum cut-offs indicate the lowest acceptable quality that VR research/clinical software should be, while the parsimonious cut-offs are offered for more robust support of the VR software’s suitability, which may be required in experimental and clinical designs with more conservative standards. However, the individual scores from the VRNQ may be modulated by individual differences and preferences unrelated to the quality of the software (Kortum and Peres, 2014). In addition, the VRNQ produces ordinal data; therefore, the median is the appropriate measure for their analysis (Harpe, 2015). Hence, the median VRNQ scores for the whole sample should be used to assess the VR software’s quality effectively. Also, the medians of the VRNQ total score and sub-scores allow the generalization of the results and comparison between different VR software (Kortum and Peres, 2014; Harpe, 2015). Researchers, clinicians, and/or research software developers should use the medians of the VRNQ total score and sub-scores to assess whether the implemented VR software exceed the minimum or parsimonious cut-offs. Hence, if the medians of the VRNQ sub-scores and totals score for VR research software meet the minimum cut-offs, then these results support the VR software’s suitability. Likewise, if the medians of VRNQ sub-scores and totals score for VR research software meet the parsimonious cut-offs, then these results provide even stronger support for its suitability. However, median scores below these cut-offs suggest that the suitability of the VR software is questionable, but they do not indicate that this VR software is certainly unsuitable.

Also, VRNQ appears as an appropriate tool to measure both VRISE and VR software features compared to other questionnaires. The SSQ is the most implemented questionnaire in VR studies. However, the SSQ only considers the symptoms pertinent to simulator sickness and it does not assess software attributes (Kennedy et al., 1993), while there is a dispute that simulator sickness symptomatology may not be the same as VRISE (Stanney et al., 1997). Alternatively, Virtual reality sickness questionnaire (VRSQ) was recently developed (Kim et al., 2018). The development of VRSQ was based on the SSQ, where the researchers attempted to isolate the items which are pertinent to VRISE (Kim et al., 2018). However, their sample size was relatively small (i.e., 24 participants ^∗^ 4 sessions = 96 observations) (Kim et al., 2018). Notably, the factor analyses of Kim et al. (2018) accepted only items pertinent to oculomotor and disorientation components of SSQ, and rejected all the items pertinent to nausea (i.e., 7 items) (Kim et al., 2018), while nausea is the most frequent symptom in VRISE (Stanney et al., 1997; Sharples et al., 2008; Bohil et al., 2011; de França and Soares, 2017; Palmisano et al., 2017). Also, comparable to SSQ, VRSQ does not consider software features. Hence, the VRNQ appears to be the only valid and suitable tool to evaluate both the intensity of predominant VRISE and the quality of VR software features.

The VRNQ allows researchers to report the quality of VR software and/or the intensity of VRISE in their VR studies. However, an in-depth assessment of the numerous software features requires a questionnaire with more than the 20 questions of the VRNQ (Zarour et al., 2015). For an in-depth software analysis, questionnaires with more questions pertinent to the whole spectrum of software features should be preferred (Zarour et al., 2015). Additionally, the VRNQ has solely five items pertinent to VRISE. Hence, it does not offer an exhaustive assessment of VRISE. Studies that aim to investigate VRISE in depth should opt for a tool which contains more items pertinent to VRISE than VRNQ (e.g., SSQ). The VNRQ is a brief questionnaire (5–10 min administration time) including 20 items, which enables researchers, clinicians, and research software developers to evaluate and report the quality of the VR software and the intensity of VRISE for research and clinical purposes.

### Maximum Duration of VR Sessions

The duration of the VR session is a crucial factor in research and/or clinical design. In our sample, the participants discontinued the VR session due to loss of interest, while none discontinued due to VRISE. In the 1st session, gamers spent significantly more time immersed than the non-gamers; a difference which modulated the difference between the two groups in the summed duration across all sessions. However, it is worth noting that there was not a significant difference between the two groups in the time spent in VR for the 2nd and 3rd sessions. The observed difference in the 1st session and the absence of a difference in the later sessions’ durations postulates that when users are familiarized with the VR technology, while the influence of their gaming experience on the session’s duration becomes insignificant. In support of this, a recent study showed that user gaming experience does not affect the perceived workload of the users in VR (Lum et al., 2018). Hence, the level of familiarization of the participants with the VR technology appears to affect substantially the duration of the VR session.

Nevertheless, in the whole sample, irrespective of participants’ gaming experience, the durations of the 2nd and 3rd sessions are sufficiently longer than the duration of the 1st session. The duration of the 3rd session is not significantly longer than the duration of the 2nd session. Furthermore, given that in each session, a different VR software was administered, the VRNQ correspondingly pinpointed significant differences amongst the implemented VR software’ quality. All the VRNQ scores for the 3rd session’s VR software are greater than the 2nd session’s VR software scores. Similarly, all the VRNQ scores for the 2nd session’s VR software are greater than the 1st session’s VR software scores. Also, the duration of VR session was positively correlated with the total score of VRNQ. Thus, the quality of the VR software as measured by the VRNQ seems to be significantly associated with the duration of the VR session.

Overall, in every session, the intensity of VRISE was reported as very mild to absent by the vast majority of the sample. However, comparable to the rest of the VRNQ scores, the VRISE score for the 3rd VR session was significantly higher (i.e., milder feeling) than the 2nd and 3rd sessions. Similarly, the VRISE score for the 2nd session’s VR software was substantially higher than the 1st session’s VR software score. Notably, there was not any difference between gamers and non-gamers in the VRNQ scores across the three sessions. Equally, the age and education of participants did not correlate with any of the VRNQ scores or the duration of sessions. Thus, the age, education, and gaming experience of the participants did not affect the responses in the VRNQ. Therefore, the observed differences in the VRISE scores between the VR sessions support that the quality of the VR software as measured by the VRNQ and the level of familiarization of the participants with the VR technology also affect the intensity of VRISE.

The findings postulate that the implementation of VR software with a maximum duration between 55 and 70 min is substantially feasible. However, long exposures in VR have been found to increase the probability of experiencing VRISE and the intensity of VRISE (Sharples et al., 2008). In our sample, especially in the 3rd session, which was substantially longer than the other sessions, the intensity of VRISE was significantly lower than the rest of the sessions. As discussed above, the substantially lower intensity of VRISE in the 3rd session appears to be a result of increased VR familiarity, and the better quality of the implemented VR software as measured by the VRNQ. Hence, researchers and/or clinicians should consider the quality of their VR software to define the appropriate duration of their VR session. In research and clinical designs where the duration of the VR session is required to be between 55 and 70 min, the researchers and/or clinicians should opt for the parsimonious cut-offs of the VRNQ to ensure adequate quality of their VR software to facilitate longer sessions without significant VRISE. Additionally, an extended introductory tutorial which allows participants to familiarize themselves with the VR technology and mechanics would assist with the implementation of longer (i.e., 55–70 min) VR sessions, where the presence and intensity of VRISE would not be significant.

### The Quality of VR Software and VRISE

The VRISE score substantially correlated with almost every item under the section of user experience and in-game assistance (see Table 6). However, the VRISE score did not correlate with VR tech (the item under the user experience’s domain) or most of the items under the section of game mechanics. The quality of VR hardware (i.e., the HMD and its controllers) and interactions (i.e., ergonomic or non-ergonomic) with the virtual environment are crucial for the alleviation or evasion of VRISE (Kourtesis et al., 2019). Nevertheless, in this sample, the VR tech item (i.e., the quality of the internal and external VR hardware) was not expected to correlate with the VRISE score, because the HMD and its 6DoF controllers were the same for all 3 VR software versions and sessions. Hence, the variance in the responses to this item was limited. Also, the three VR software games share common game mechanics, especially the same navigation system (i.e., teleportation) and a similar amount of physical mobility. Likewise, apart from some controls (i.e., the button to grab items), the interaction systems of the implemented VR software were very proximal. Therefore, the absence of a correlation between VRISE scores and most of the items in the game mechanics’ section was also an expected outcome. Nonetheless, the VRISE score was strongly associated with the level of immersion and enjoyment, the quality of graphics and sound, the comfort to pick and place 3D objects, and the usefulness of in-game assistance modes (i.e., tutorials, instructions, and prompts).

The items which correlated with the VRISE score were also included in the best models of predicting its value (see Table 7). Importantly, the best model includes as predictors of VRISE, the level of immersion, the quality of graphics and sound, and the helpfulness of in-game instructions and prompts (see Table 7). The higher scores for prompts and instructions indicate that the user was substantially assisted by the in-game assistance (e.g., an arrow showing the direction that the user should follow) to orientate and guide his or herself from one point of interest to the next in accordance with the scenario of the VR experience. This may be interpreted as ease to orient and interact with the virtual environment, as well as a significant decrease in confusion (Brade et al., 2018). The quality of the in-game assistance methods is essential for the usability and enjoyment that VR software offers (Brade et al., 2018). Equally, the quality of the graphics is predominantly dependent upon rendering which encompasses the in-game quality of the image known as perceptual quality, and the exclusion of redundant visual information known as occlusion culling (Lavoué and Mantiuk, 2015). The improvement of these two factors not only results in improved quality of the graphics but also in improved performance of the software (Brennesholtz, 2018). Furthermore, the spatialized sound of VR software, which assists the user to orient his or herself (Ferrand et al., 2017), deepens the experienced immersion (Riecke et al., 2011), and enriches the geometry of the virtual space without affecting the performance of the software (Kobayashi et al., 2015). Lastly, the level of immersion appears to be negatively correlated with the frequency and intensity of VRISE (Milleville-Pennel and Charron, 2015; Weech et al., 2019). The best model hence aligns with the relevant literature and provides further evidence in support of the utility of the VRNQ as a valid and efficient tool to appraise the quality of the VR software and intensity of VRISE.

### Limitations and Future Studies

This study also has some limitations. In this study, construct validity for the VRNQ is provided. However, future work should endeavor to provide convergent validation of the VRNQ with tools that measure VRISE symptomatology (e.g., SSQ) and/or VR software attributes. Moreover, the sample size was relatively small, but it offered an adequate statistical power for the conducted analyses. Also, the VRNQ does not directly quantify linear or angular accelerations, which may induce intense VRISE in a relatively short period of time (McCauley and Sharkey, 1992; LaViola, 2000; Gavgani et al., 2018). However, the VRNQ quantifies the effect(s) of linear and angular accelerations (i.e., VRISE), where VR software with a highly provocative content (e.g., linear and angular accelerations) would fail to meet or exceed the VRNQ cut-offs for the VRISE domain. Furthermore, the study utilized only one type of VR hardware, which did not allow us to inspect the effect of VR HMD’s quality on VRISE presence and intensity. Similarly, our VR software did not allow us to compare different ergonomic interactions or levels of provocative potency pertaining to VRISE. Future studies with a larger sample, various types of VR hardware, and VR software with substantially more diverse features will offer further insights on the impact of software features on VRISE intensity, as well as provide additional support for the VRNQ’s structural model. Lastly, neuroimaging (e.g., electroencephalography) and physiological data (e.g., heart rates) may correlate, classify, and predict VRISE symptomatology (Kim et al., 2005; Dennison et al., 2016, 2019). Hence, future studies should consider collecting neuroimaging and/or physiological data that could further elucidate the relationship between VRNQ’s VRISE score(s) and brain region activation or cardiovascular responses (e.g., heart rate).

## Conclusion

This study showed that the VRNQ is a valid and reliable tool which assesses the quality of VR software and intensity of VRISE. Our findings support the viability of VR sessions with a duration up to 70 min, when the participants are familiarized with VR tech through an induction session, and the quality of the VR software meets the parsimonious cut-offs of VRNQ. Also, our results offered insights on the software-related predictors of VRISE intensity, such as the level of immersion, the quality of graphics and sound, and the helpfulness of in-game instructions and prompts. Finally, the VRNQ enables researchers to quantitatively assess and report the quality of VR software features and intensity of VRISE, which are vital for the efficacious implementation of immersive VR systems in cognitive neuroscience and neuropsychology. The minimum and parsimonious cut-offs of VRNQ may appraise the suitability of VR software for implementation in research and clinical settings. The VRNQ and the findings of this study contribute to the endeavor of establishing thorough VR research and clinical methods that are crucial to guarantee the viability of implementing immersive VR systems in cognitive neuroscience and neuropsychology.

## Data Availability Statement

The datasets generated for this study are available on request to the corresponding author.

## Ethics Statement

The studies involving human participants were reviewed and approved by Philosophy, Psychology and Language Sciences Research Ethics Committee of the University of Edinburgh. The patients/participants provided their written informed consent to participate in this study.

## Author Contributions

PK had the initial idea and contributed to every aspect of this study. SC, LD, and SM contributed to the methodological aspects and the discussion of the results. The VRNQ may be downloaded from Supplementary Material.

## Conflict of Interest

The authors declare that the research was conducted in the absence of any commercial or financial relationships that could be construed as a potential conflict of interest.

## Footnotes

1 https://store.steampowered.com/app/448280/Job_Simulator/

2 https://store.steampowered.com/app/450390/The_Lab/

3 https://store.steampowered.com/app/469610/Rick_and_Morty_Virtual_Rickality/

## References

[B1] Arafat I. M. Ferdous S. M. S. Quarles J. (2018). “Cybersickness-provoking virtual reality alters brain signals of persons with multiple sclerosis,” in *2018 IEEE Conference on Virtual Reality and 3D User Interfaces (VR)*, (Reutlingen: IEEE), 1–120.

[B2] Arbuckle J. L. (2014). *Amos (Version 23.0).* Chicago: IBM SPSS.

[B3] Bohil C. J. Alicea B. Biocca F. A. (2011). Virtual reality in neuroscience research and therapy. *Nat. Rev. Neurosci.* 12 752–762. 10.1038/nrn3122 22048061

[B4] Brade J. Dudczig M. Klimant P. (2018). “Using virtual prototyping technologies to evaluate human-machine-interaction concepts,” in *aw&I Conference*, Vol. 3 (Chemnitz).

[B5] Brennesholtz M. S. (2018). 3−1: invited paper: vr standards and guidelines. *SID Symposium Dig. Tech. Pap.* 49 1–4. 10.1002/sdtp.12476

[B6] Button K. S. Ioannidis J. P. Mokrysz C. Nosek B. A. Flint J. Robinson E. S. (2013). Confidence and precision increase with high statistical power. *Nat. Rev. Neurosci.* 14:585. 10.1038/nrn3475-c4 23820778

[B7] Cohen J. (2013). *Statistical Power Analysis for the Behavioral Sciences*. New York, NY: Routledge.

[B8] Cole D. A. (1987). Utility of confirmatory factor analysis in test validation research. *J. Consul. Clin. Psychol.* 55 584–594. 10.1037//0022-006x.55.4.584 3624616

[B9] de França A. C. P. Soares M. M. (2017). “Review of virtual reality technology: an ergonomic approach and current challenges,” in *International Conference on Applied Human Factors and Ergonomics*, (Cham: Springer), 52–61. 10.1007/978-3-319-60582-1_6

[B10] Dennison M. Jr. D’Zmura M. Harrison A. Lee M. Raglin A. (2019). “Improving motion sickness severity classification through multi-modal data fusion,” in *Artificial Intelligence and Machine Learning for Multi-Domain Operations Applications*, Vol. 11006 (Baltimore, MA.), 110060T.

[B11] Dennison M. S. Wisti A. Z. D’Zmura M. (2016). Use of physiological signals to predict cybersickness. *Displays* 44 42–52. 10.1016/j.displa.2016.07.002

[B12] Ferrand S. Alouges F. Aussal M. (2017). “Binaural spatialization methods for indoor navigation,” in *Audio Engineering Society Convention*, Vol. 142 (New York, NY: Audio Engineering Society).

[B13] Gavgani A. M. Wong R. H. Howe P. R. Hodgson D. M. Walker F. R. Nalivaiko E. (2018). Cybersickness-related changes in brain hemodynamics: a pilot study comparing transcranial Doppler and near-infrared spectroscopy assessments during a virtual ride on a roller coaster. *Physiol. Behav.* 191 56–64. 10.1016/j.physbeh.2018.04.007 29649509

[B14] Harpe S. E. (2015). How to analyze likert and other rating scale data. *Curr. Pharm. Teach. Learn.* 7 836–850. 10.1016/j.cptl.2015.08.001

[B15] Hopwood C. J. Donnellan M. B. (2010). How should the internal structure of personality inventories be evaluated? *Personal. Soc. Psychol. Rev.* 14 332–346. 10.1177/1088868310361240 20435808

[B16] Hu L. T. Bentler P. M. (1999). Cutoff criteria for fit indexes in covariance structure analysis: conventional criteria versus new alternatives. *Struc. Equ. Model.* 6 1–55. 10.1080/10705519909540118

[B17] Ibm Corp, (2016). *IBM SPSS Statistics for Windows, Version 24.0.* Armonk, NY: IBM Corp.

[B18] Jackson D. L. Gillaspy J. A. Jr. Purc-Stephenson R. (2009). Reporting practices in confirmatory factor analysis: an overview and some recommendations. *Psychol. Methods* 14 6–23. 10.1037/a0014694 19271845

[B19] Jasp Team, (2017). *JASP (Version 0.8.*1.2).

[B20] Kennedy R. S. Lane N. E. Berbaum K. S. Lilienthal M. G. (1993). Simulator sickness questionnaire: an enhanced method for quantifying simulator sickness. *Int. J. Aviat. Psychol.* 3 203–220. 10.1207/s15327108ijap0303_3

[B21] Kim H. K. Park J. Choi Y. Choe M. (2018). Virtual reality sickness questionnaire (VRSQ): motion sickness measurement index in a virtual reality environment. *Appl. Ergon.* 69 66–73. 10.1016/j.apergo.2017.12.016 29477332

[B22] Kim Y. Y. Kim H. J. Kim E. N. Ko H. D. Kim H. T. (2005). Characteristic changes in the physiological components of cybersickness. *Psychophysiology* 42 616–625. 16176385 10.1111/j.1469-8986.2005.00349.x

[B23] Kobayashi M. Ueno K. Ise S. (2015). The effects of spatialized sounds on the sense of presence in auditory virtual environments: a psychological and physiological study. *Presence* 24 163–174. 10.1162/pres_a_00226

[B24] Kortum P. Peres S. C. (2014). The relationship between system effectiveness and subjective usability scores using the system usability scale. *Int. J. Hum. Comput. Int.* 30 575–584. 10.1080/10447318.2014.904177

[B25] Kourtesis P. Collina S. Doumas L. A. A. MacPherson S. E. (2019). Technological competence is a precondition for effective implementation of virtual reality head mounted displays in human neuroscience: a technological review and meta-analysis. *Front. Hum. Neurosci.* 13:342. 10.3389/fnhum.2019.00342 PMC6783565 31632256

[B26] LaViola J. J. Jr. (2000). A discussion of cybersickness in virtual environments. *ACM Sigchi Bull.* 32 47–56. 10.1145/333329.333344

[B27] Lavoué G. Mantiuk R. (2015). “Quality assessment in computer graphics,” in *Visual Signal Quality Assessment*, (Cham: Springer), 243–286. 10.1007/978-3-319-10368-6_9

[B28] Liang F. Paulo R. Molina G. Clyde M. A. Berger J. O. (2008). Mixtures of g priors for bayesian variable selection. *J. Am. Stat. Assoc.* 103 410–423.

[B29] Lum H. C. Greatbatch R. Waldfogle G. Benedict J. (2018). “How Immersion, Presence, Emotion, & Workload Differ in Virtual Reality and Traditional Game Mediums,” in *Proceedings of the Human Factors and Ergonomics Society Annual Meeting*, Vol. 62 (Los Angeles, CA: SAGE Publications), 1474–1478. 10.1177/1541931218621334

[B30] Marsman M. Wagenmakers E. J. (2017). Bayesian benefits with JASP. *Eur. J. Dev. Psychol.* 14 545–555. 10.1080/17405629.2016.1259614

[B31] McCauley M. E. Sharkey T. J. (1992). Cybersickness: perception of self-motion in virtual environments. *Presence* 1 311–318. 10.1162/pres.1992.1.3.311

[B32] Milleville-Pennel I. Charron C. (2015). Do mental workload and presence experienced when driving a real car predispose drivers to simulator sickness? An exploratory study. *Accid. Anal. Prev.* 74 192–202. 10.1016/j.aap.2014.10.021 25463960

[B33] Mittelstaedt J. Wacker J. Stelling D. (2018). Effects of display type and motion control on cybersickness in a virtual bike simulator. *Displays* 51 43–50. 10.1016/j.displa.2018.01.002

[B34] Mittelstaedt J. M. Wacker J. Stelling D. (2019). VR aftereffect and the relation of cybersickness and cognitive performance. *Vir. Real.* 23 143–154. 10.1007/s10055-018-0370-3

[B35] Nalivaiko E. Davis S. L. Blackmore K. L. Vakulin A. Nesbitt K. V. (2015). Cybersickness provoked by head-mounted display affects cutaneous vascular tone, heart rate and reaction time. *Physiol. Behav.* 151 583–590. 10.1016/j.physbeh.2015.08.043 26340855

[B36] Nesbitt K. Davis S. Blackmore K. Nalivaiko E. (2017). Correlating reaction time and nausea measures with traditional measures of cybersickness. *Displays* 48 1–8. 10.1016/j.displa.2017.01.002

[B37] Nunally J. C. Bernstein I. H. (1994). *Psychometric Theory*, 3rd Edn New Yokr, NY: Mcgraw-Hill.

[B38] Palmisano S. Mursic R. Kim J. (2017). Vection and cybersickness generated by head-and-display motion in the oculus rift. *Displays* 46 1–8. 10.1016/j.displa.2016.11.001

[B39] Parsons T. D. (2015). Virtual reality for enhanced ecological validity and experimental control in the clinical, affective and social neurosciences. *Front. Hum. Neurosci.* 9:660. 10.3389/fnhum.2015.00660 26696869 PMC4675850

[B40] Parsons T. D. McMahan T. Kane R. (2018). Practice parameters facilitating adoption of advanced technologies for enhancing neuropsychological assessment paradigms. *Clin. Neuropsychol.* 32 16–41. 10.1080/13854046.2017.1337932 28590154

[B41] Riecke B. E. Feuereissen D. Rieser J. J. McNamara T. P. (2011). “Spatialized sound enhances biomechanically-induced self-motion illusion (vection),” in *Proceedings of the SIGCssHI Conference on Human Factors in Computing Systems*, (New York, NY: ACM.), 2799–2802.

[B42] Rouder J. N. Morey R. D. (2012). Default bayes factors for model selection in regression. *Multivar. Behav. Res.* 47 877–903. 10.1080/00273171.2012.734737 26735007

[B43] Sharples S. Cobb S. Moody A. Wilson J. R. (2008). Virtual reality induced symptoms and effects (VRISE): Comparison of head mounted display (HMD), desktop and projection display systems. *Displays* 29 58–69. 10.1016/j.displa.2007.09.005

[B44] Soper D. S. (2019a). *A-priori Sample Size Calculator for Structural Equation Models.* Available at http://www.danielsoper.com/statcalc (accessed July 10, 2019).

[B45] Soper D. S. (2019b). *Post-hoc Statistical Power Calculator for Multiple Regression.* Available at http://www.danielsoper.com/statcalc (accessed July 10, 2019).

[B46] Stanney K. M. Kennedy R. S. Drexler J. M. (1997). “Cybersickness is not simulator sickness,” In *Proceedings of the Human Factors and Ergonomics Society annual meeting*, (Los Angeles, CA: SAGE Publications). 41 1138–1142.

[B47] Toschi N. Kim J. Sclocco R. Duggento A. Barbieri R. Kuo B. (2017). Motion sickness increases functional connectivity between visual motion and nausea-associated brain regions. *Auton. Neurosci.* 202 108–113. 10.1016/j.autneu.2016.10.003 28245927 PMC5332554

[B48] Weech S. Kenny S. Barnett-Cowan M. (2019). Presence and cybersickness in virtual reality are negatively related: a review. *Front. Psychol.* 10:158. 10.3389/fpsyg.2019.00158 30778320 PMC6369189

[B49] Wetzels R. Wagenmakers E. J. (2012). A default bayesian hypothesis test for correlations and partial correlations. *Psychon. Bull. Rev.* 19 1057–1064. 10.3758/s13423-012-0295-x 22798023 PMC3505519

[B50] Zarour M. Abran A. Desharnais J. M. Alarifi A. (2015). An investigation into the best practices for the successful design and implementation of lightweight software process assessment methods: a systematic literature review. *J. Syst. Softw.* 101 180–192. 10.1016/j.jss.2014.11.041

