# Supplementary material for: Validation of the Virtual Reality Neuroscience Questionnaire: Maximum Duration of Immersive Virtual Reality Sessions Without the Presence of Pertinent Adverse Symptomatology
Source: Front Hum Neurosci. 2019 Nov 26;13:417. doi: 10.3389/fnhum.2019.00417 (PMC6901952; doi:10.3389/fnhum.2019.00417)
Supplement: Supplementary file 1 [file Data_Sheet_1.PDF]

VIRTUAL REALITY NEUROSCIENCE QUESTIONNAIRE

Please, from 1 to 7, **circle** the response that closely represents your opinion.

UNIVERSITA DEGLI STUDI SUOR ORSOLA BENINCASA
SCIENZA NUOVA

THE UNIVERSITY of EDINBURGH
School of Philosophy, Psychology and Language Sciences

User Experience

What is the level of immersion you experienced?

1	2	3	4	5	6	7
**Extremely Low**	**Very Low**	**Low**	**Neutral**	**High**	**Very High**	**Extremely High**

Please write below any additional comments and/or suggestions relevant to the question above:

What was your level of enjoyment of the VR experience?

1	2	3	4	5	6	7
**Extremely Low**	**Very Low**	**Low**	**Neutral**	**High**	**Very High**	**Extremely High**

Please write below any additional comments and/or suggestions relevant to the question above:

How was the quality of the graphics?

1	2	3	4	5	6	7
**Extremely Low**	**Very Low**	**Low**	**Neutral**	**High**	**Very High**	**Extremely High**

Please write below any additional comments and/or suggestions relevant to the question above:

How was the quality of the sound?

1	2	3	4	5	6	7
**Extremely Low**	**Very Low**	**Low**	**Neutral**	**High**	**Very High**	**Extremely High**

Please write below any additional comments and/or suggestions relevant to the question above:

How was the quality of the VR technology overall (i.e. hardware & peripherals)?

1	2	3	4	5	6	7
**Extremely Low**	**Very Low**	**Low**	**Neutral**	**High**	**Very High**	**Extremely High**

Please write below any additional comments and/or suggestions relevant to the question above:

Game Mechanics

How easy was to use the navigation system (e.g. teleportation) in the virtual environment?

1	2	3	4	5	6	7
**Extremely Difficult**	**Very Difficult**	**Difficult**	**Neutral**	**Easy**	**Very Easy**	**Extremely Easy**

Please write below any additional comments and/or suggestions relevant to the question above:

How easy was to physically move in the virtual environment?

1	2	3	4	5	6	7
**Extremely Difficult**	**Very Difficult**	**Difficult**	**Neutral**	**Easy**	**Very Easy**	**Extremely Easy**

Please write below any additional comments and/or suggestions relevant to the question above:

How easy was to pick up and/or place items in the virtual environment?

1	2	3	4	5	6	7
**Extremely Difficult**	**Very Difficult**	**Difficult**	**Neutral**	**Easy**	**Very Easy**	**Extremely Easy**

Please write below any additional comments and/or suggestions relevant to the question above:

How easy was to use items in the virtual environment?

1	2	3	4	5	6	7
**Extremely Difficult**	**Very Difficult**	**Difficult**	**Neutral**	**Easy**	**Very Easy**	**Extremely Easy**

Please write below any additional comments and/or suggestions relevant to the question above:

How easy was the 2-handed interaction e.g., grab the tablet with the one hand, and push the button with the other hand?

1	2	3	4	5	6	7
**Extremely Difficult**	**Very Difficult**	**Difficult**	**Neutral**	**Easy**	**Very Easy**	**Extremely Easy**

Please write below any additional comments and/or suggestions relevant to the question above:

In-Game Assistance

How easy was to complete the tutorial(s)?

1	2	3	4	5	6	7
**Extremely Difficult**	**Very Difficult**	**Difficult**	**Neutral**	**Easy**	**Very Easy**	**Extremely Easy**

Please write below any additional comments and/or suggestions relevant to the question above:

How helpful was/were the tutorial(s)?

1	2	3	4	5	6	7
**Extremely Unhelpful**	**Very Unhelpful**	**Unhelpful**	**Neutral**	**Helpful**	**Very Helpful**	**Extremely Helpful**

Please write below any additional comments and/or suggestions relevant to the question above:

How did you feel about the duration of the tutorial(s)?

1	2	3	4	5	6	7
**Extremely More Time Needed**	**Much More Time Needed**	**More Time Needed**	**Neutral**	**Enough Time Available**	**Much Time Available**	**Plenty of Time Available**

Please write below any additional comments and/or suggestions relevant to the question above:

How helpful were the in-game instructions for the task you needed to perform?

1	2	3	4	5	6	7
**Extremely Unhelpful**	**Very Unhelpful**	**Unhelpful**	**Neutral**	**Helpful**	**Very Helpful**	**Extremely Helpful**

Please write below any additional comments and/or suggestions relevant to the question above:

How helpful were the in-game prompts e.g. arrows showing the direction, or labels?

1	2	3	4	5	6	7
**Extremely Unhelpful**	**Very Unhelpful**	**Unhelpful**	**Neutral**	**Helpful**	**Very Helpful**	**Extremely Helpful**

Please write below any additional comments and/or suggestions relevant to the question above:

VR Induced Symptoms and Effects (VRISE)

Did you experience nausea?

1	2	3	4	5	6	7
**Extremely Intense Feeling**	**Very Intense Feeling**	**Intense Feeling**	**Moderate Feeling**	**Mild Feeling**	**Very Mild Feeling**	**Absent**

Please write below any additional comments and/or suggestions relevant to the question above:

Did you experience disorientation?

1	2	3	4	5	6	7
**Extremely Intense Feeling**	**Very Intense Feeling**	**Intense Feeling**	**Moderate Feeling**	**Mild Feeling**	**Very Mild Feeling**	**Absent**

Please write below any additional comments and/or suggestions relevant to the question above:

Did you experience dizziness?

1	2	3	4	5	6	7
**Extremely Intense Feeling**	**Very Intense Feeling**	**Intense Feeling**	**Moderate Feeling**	**Mild Feeling**	**Very Mild Feeling**	**Absent**

Please write below any additional comments and/or suggestions relevant to the question above:

Did you experience fatigue?

1	2	3	4	5	6	7
**Extremely Intense Feeling**	**Very Intense Feeling**	**Intense Feeling**	**Moderate Feeling**	**Mild Feeling**	**Very Mild Feeling**	**Absent**

Please write below any additional comments and/or suggestions relevant to the question above:

Did you experience instability?

1	2	3	4	5	6	7
**Extremely Intense Feeling**	**Very Intense Feeling**	**Intense Feeling**	**Moderate Feeling**	**Mild Feeling**	**Very Mild Feeling**	**Absent**

Please write below any additional comments and/or suggestions relevant to the question above:

Virtual Reality Neuroscience Questionnaire (VRNQ) – Scores

Section	Score	Minimum Cut-offs	Parsimonious Cut-offs
User Experience		≥ 25	≥ 30
Game Mechanics		≥ 25	≥ 30
In-Game Assistance		≥ 25	≥ 30
VRISE		≥ 25	≥ 30
Total VRNQ		≥ 100	≥ 120

The median of each sub-score and totals score should meet the suggested cut-offs to support that the evaluated VR software has an adequate quality without any significant VRISE.
The utilisation of the parsimonious cut-offs more robustly supports the suitability of the VR software.

The VR Neuroscience Questionnaire (VRNQ) was developed by Panagiotis Kourtesis

in affiliation with University of Edinburgh & University Suor Orsola Benincasa of Naples.

SCIENZA NUOVA
UNIVERSITA DEGLI STUDI SUOR ORSOLA BENINCASA

THE UNIVERSITY of EDINBURGH
School of Philosophy, Psychology and Language Sciences
